# Prevalence of Insomnia and Sleep Habits during the First and Second Wave of COVID-19 in Belgium

**DOI:** 10.5334/pb.1160

**Published:** 2023-02-21

**Authors:** Aurore Roland, Clara Colomb, Stéphane Noël, Arcady Putilov, Halszka Oginska, Bérénice Delwiche, Oumaima Benkirane, Maxime Windal, Nathalie Vanlaer, Giovanni Briganti, Judith Carrasquer-Ferrer, Behrouz Riahi, Charles Konreich, Daniel Neu, Johan Newell, Olivier Vermylen, Philippe Peigneux, Nathalie Pattyn, Johan Verbraecken, Ilse De Volder, Tim Vantilborgh, Joeri Hofmans, Martine Van Puyvelde, Olivier Mairesse

**Affiliations:** 1 Vrije Universiteit Brussel, Belgium; 2 Fonds Wetenschappelijk Onderzoek, Belgium; 3 CHU Brugmann, Belgium; 4 CHU Charleroi, Belgium; 5 Russian Academy of Sciences, Research Institute for Molecular Biology and Biophysics of the Federal Research Centre for Fundamental and Translational Medicine, Russia; 6 Jagiellonian University, Poland; 7 UZ Brussel, Belgium; 8 Université libre de Bruxelles, Belgium; 9 Université de Mons, Belgium; 10 CHIREC, Belgium; 11 Royal Military Academy, Belgium; 12 Universiteit Antwerpen, Belgium; 13 UZA, Belgium

**Keywords:** insomnia, sleep habits, COVID-19, pandemic, sleep

## Abstract

Belgium has one of the highest numbers of COVID-19 cases per 1 million inhabitants. The pandemic has led to significant societal changes with repercussions on sleep and on mental health. We aimed to investigate the effect of the first and the second wave of COVID-19 on the sleep of the Belgian populationWe launched two online questionnaires, one during the first lockdown (7240 respondents) and one during the second (3240 respondents), to test differences in self-reported clinical insomnia (as measured by the Insomnia Severity Index) and sleep habits during the two lockdowns in comparison with the pre-COVID period. The number of persons with clinical insomnia rose during the first lockdown (19.22%) and further during the second (28.91%) in comparison with pre-lockdown (7.04–7.66%). Bed and rise times were delayed and there was an increased time in bed and sleep onset latency. There was further a decrease in total sleep time and in sleep efficiency during both confinements. The prevalence of clinical insomnia quadrupled during the second wave in comparison with the pre-lockdown situation. Sleep habits were most altered in the younger population, indicating a greater risk for this group to develop a sleep-wake rhythm disorder.

## Introduction

Since its first appearance at the end of December 2019 in the Chinese city of Wuhan, the spread of the severe acute respiratory syndrome coronavirus 2 (SARS-CoV-2/COVID-19) has been declared a worldwide pandemic, threatening the lives of millions around the globe (Tang et al., 2020). By March 15, 2022, Belgium was the 29^th^ country in the world with the highest number of cases per 1 million inhabitants (Worldometer, 2022). As of the 17^th^ of December 2021, 1.40% of persons in Belgium with a confirmed COVID-19 infection died of this disease (World Health Organization, 2021). Aside from the well-documented somatic symptoms often associated with a confirmed infection, such as fever and respiratory distress (Pascarella et al., 2020), the psychological distress was high during the pandemic and related to sleep problems (Alimoradi et al., 2021; Huang et al., 2022; Zhang et al., 2022).

Before the COVID-19 pandemic, the lifetime prevalence of insomnia as a symptom exceeded 90%. Acute insomnia (between 3 days and 3 months) affected 30 to 35% of the general population, whereas the prevalence of chronic insomnia (≥3 months) at syndrome level according to International Classification of Sleep Disorders -3^rd^ edition (ICSD-3) criteria was estimated at 10% (de Valck et al., 2019). Generally, the combination of both predisposing biopsychosocial factors (such as education or environment) and actual precipitating factors (such as lifetime events and somatic or psychiatric illnesses) contribute to inter-individual differences in the appearance of classic insomnia symptoms, such as difficulties falling asleep, difficulties staying asleep, and/or early morning awakenings (Perlis et al., 2011).

So far, four studies tested the impact of the first lockdown on clinical insomnia (as measured by the ISI) in the general population (Kocevska et al., 2020; Li et al., 2020; Lin et al., 2021; Marelli et al., 2021). Three of these studies showed a significant increase in the number of people suffering from clinical insomnia (Li et al., 2020; Lin et al., 2021; Marelli et al., 2021). Kocevska et al. (2020) found that most individuals who slept well before the pandemic saw their sleep worsened during the confinement. However, a quarter of the people who suffered from clinical insomnia before the pandemic reported an improvement of their sleep during the lockdown. These changes were associated with experiencing negative emotions. Worsened ISI-scores were further associated with sociodemographic variables such as being female (Li et al., 2020; Marelli et al., 2021) and a younger age (Marelli et al., 2021).

Regarding more general sleep-related behaviors, most studies report a delay in BT and RT during the first lockdown (Cellini et al., 2020, 2021; Gupta et al., 2020; Li et al., 2020; Majumdar et al., 2020; Marelli et al., 2021; Sinha et al., 2020; Wright et al., 2020). The change in BT and RT was more pronounced in younger people (Cellini et al., 2021; Leone et al., 2020) and for females (Sinha et al., 2020). This delay in BT and RT usually meant an increase in TIB (Cellini et al., 2020, 2021; Li et al., 2020; Robillard et al., 2021), which was greater among younger persons in the study of Cellini et al (Cellini et al., 2021). Both an increase (Blume et al., 2020; Cellini et al., 2021; Leone et al., 2020; Li et al., 2020; Sinha et al., 2020) and a decrease (Gupta et al., 2020; López-Moreno et al., 2020; Robillard et al., 2021) in TST between pre- and peri-lockdown has been reported. A lower TST during lockdown was related to the male gender (Sinha et al., 2020) and an older age (Leone et al., 2020; López-Moreno et al., 2020; Marelli et al., 2021; Sinha et al., 2020). Li et al. (2020) reported that this increase in TST and TIB was found alongside a decrease in SE. These results are in line with a reported increase in difficulties falling asleep (López-Moreno et al., 2020; Marelli et al., 2021) and in sleep onset latency (SOL) during the confinement (Cellini et al., 2021; Gupta et al., 2020; Marelli et al., 2021; Robillard et al., 2021).

So far, only two studies investigated the effect of the second confinement on sleep. Salfi et al. (2021) compared clinical insomnia, BT, RT, TIB, TST, SOL, SE and sleep quality during the second lockdown in comparison with the first. They found an earlier BT and RT, a higher sleep quality and a lower SOL, TST and ISI-score during the second confinement compared to the first. Conte et al. (2021) investigated the effect of the first and second confinements on BT, RT, TIB, SOL and sleep quality. They found that BT and RT were delayed during the first lockdown and returned to their pre-COVID times during the second. SOL and TIB both increased during the first lockdown and returned to their pre-COVID levels during the second.

The purpose of this study was to investigate the effect of the first and second confinement on the prevalence of insomnia symptoms and on self-reported sleep parameters in the Belgian population. Our hypotheses were:

1. We expected an increase in clinical insomnia prevalence during the first and second lockdown compared to before the lockdown.
2. During the first lockdown, we expected a significant delay in BT and RT, an increase in TIB and SOL and a decrease in TST and SE. During the second lockdown, we expected these variables to return to their pre-pandemic values.

To the best of our knowledge, this study is the first to investigate both the effect of the first and second confinement on clinical insomnia and sleep parameters (BT, RT, TIB, TST, SOL and SE) each in comparison with the pre-lockdown situation in the Belgian population. Considering the above-mentioned link between mental health and sleep problems during the pandemic (Alimoradi et al., 2021; Huang et al., 2022; Zhang et al., 2022), documenting an increasing insomnia prevalence in Belgium could be an indication of the burden of the pandemic on the mental health of the Belgian population. It is also an indication of the impact on social security (Léger & Bayon, 2010), the numbers needed to treat and insomnia is a predictive factor for the development of depression, anxiety, alcohol abuse and psychosis (Hertenstein et al., 2019).

## Materials and Methods

### Participants

In total, 7240 persons responded to questionnaire of the first lockdown. Mean age was 41.20 years (*SD* = 15.02). Male participants were significantly older than the female participants (*t*(5917) = 13.71, *p* =< .001, *d* = 0.38). The majority of participants were female (67.29%) and there was a significant difference in the gender proportions between age categories (*F*(3, 5116) = 51.69, *p* =< .001, *η^2^_p_* = .03). Because participants were pseudonymized, some participants may have answered both questionnaires.

3240 respondents responded to the second questionnaire of the second lockdown. Mean age was 41.33 years (*SD* = 14.37). Once again, male participants were significantly older than female participants (*t*(2835) = 6.52, *p* =< .001, *d* = 0.30). The majority of respondents were female (68.01%) and there was, also in the second data collection, a significant difference in the gender proportions between age categories (*F*(3, 2095) = 13.12, p =< .001, *η^2^_p_* = .02).

### Materials and procedure

The first questionnaire, available in ten languages (French, Dutch, English, German, Spanish, Swedish, Russian, Portuguese, Italian and Polish) was launched online on the 1^st^ of April 2020 and distributed via hospital newsletters, radio, social media, the websites of the Belgian Association of Sleep research and Sleep medicine (BASS), European Sleep Research Society (ESRS) and of the World Sleep Society and international and local newspapers. The last responses to be accepted were from May 22^nd^, 2020. Since most respondents replied to the Dutch (53.7%) and the French (29.4%) versions, we opted for a rollout of only these two versions. The second questionnaire (only in French and Dutch) was launched on the 23^rd^ of November 2020, distributed via hospital newsletters, regular media (newspapers and radio) and social media. Data were retrieved on the 6^th^ of February 2021. Most respondents replied to the Dutch version (75.6%). This study was approved by a local Committee for Medical Ethics (P20/38_29/04; CCB: B3252020000020).

The first questionnaire comprised 50 questions of which the following were used in this study: BT, SOL, RT and TST since the pandemic (questions 15–18). Participants were next instructed to reply to the seven questions of the Insomnia Severity Index (ISI) (Morin, 1993) (questions 19–25). These questions measure seven domains: severity of sleep onset, sleep maintenance and early morning wakening problems, sleep dissatisfaction, interference of sleep difficulties with daytime functioning, noticeability of sleep problems by others and distress caused by the sleep difficulties. Each question was scored on a 5-point Likert scale (0 = no problem; 4 = very severe problem) and the scores were then added up. A total score between 0 and 7 signifies absence of clinical insomnia, a score between 8 and 14 a sub-clinical insomnia, between 15 and 21 a moderate clinical insomnia and between 21 and 28 a severe clinical insomnia. In our study, participants were considered having insomnia when they scored 15 or more on the ISI. The ISI is a reliable and valid instrument to measure insomnia with an average item-total correlation of 0.56 and correlations with sleep diaries ranging from r = .32 to r = .55 (Bastien, 2001). Respondents were instructed to complete the questionnaire with respect to how they felt during the past two weeks (Morin, 1993). However, for the purpose of the current study, instructions were modified and participants were asked to report how they felt since the start of the lockdown. Then, questions 15 to 25 were repeated, but this time to be answered with the pre-COVID-19 situation in mind (questions 33–43).

The second questionnaire comprised 74 questions, similar to the first questionnaire, but this time aimed at comparing the second confinement with the pre-lockdown situation.

### Statistical analysis

Group differences (gender, age categories, …) were investigated using independent *t* tests and multivariate analyses of variance (MANOVA). Within subject differences (e.g., pre vs peri lockdown) were analyzed using paired sample *t* tests. All variables showed univariate and multivariate normality. To investigate if there was a change in the proportions of the different ISI categories we used the Stuart-Maxwell test, an extension of the Chi² square test for n × n matrices with non-orthogonal data (repeated measures). All statistical analyses were performed using SPSS version 27 (https://www.ibm.com/products/spss-statistics) and JASP version 0.14.1.0. (https://jasp-stats.org/).

## Results

### Descriptives

**Figure 1:**
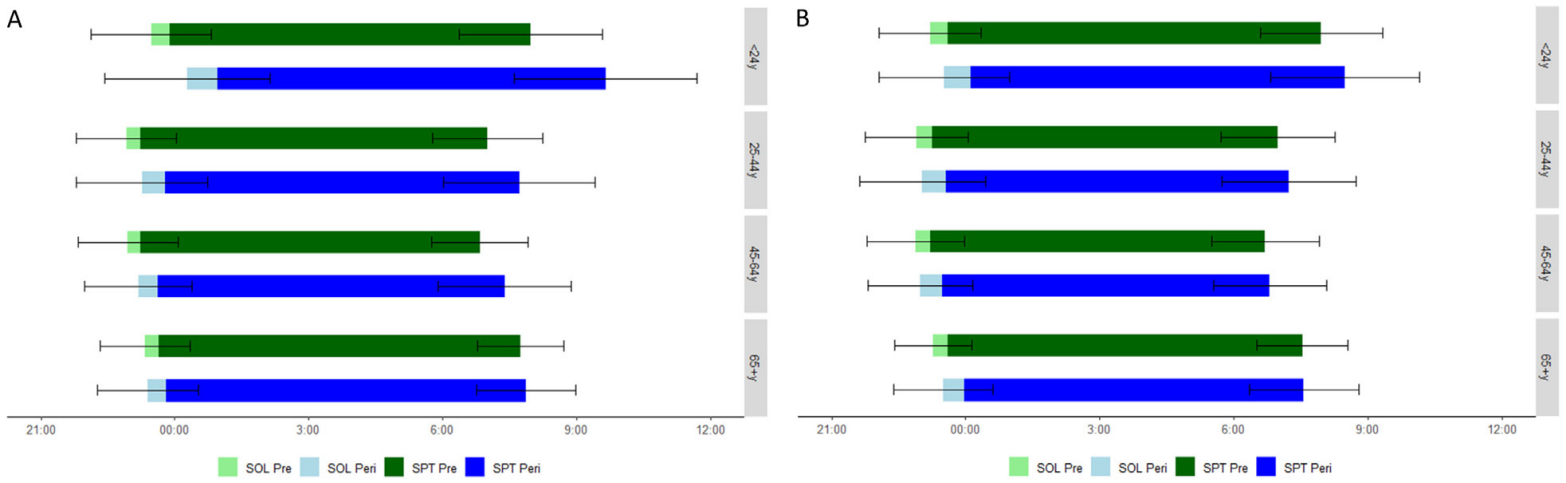
Age Differences in Bed and Rise Times. *Note*: y = years; pre = pre-lockdown; peri = peri-lockdown; SOL = sleep onset latency; SPT = sleep period time (A) Results of the first questionnaire comparing the pre-covid situation with the first lockdown. (B) Results of the second questionnaire comparing the pre-covid situation with the second lockdown.

Less than one percent (0.93%) of the participants of our first questionnaire had been diagnosed with COVID-19. Descriptive data for the first wave stratified for sex can be found in Table 1 and data stratified for age categories in Table 3. During the second wave, 7.58% of the respondents had already been diagnosed COVID-19 positive. Descriptive data for the second wave can be found in Table 2 and Table 4. There were significant sex differences in most descriptive variables, but effect sizes were either small or negligible. For age, there were also significant differences on all descriptive variables, but again most effect sizes were relatively small considering non-contextual benchmarks [27]. A few were moderate and RT peri-lockdown during the first wave was the only one with a large effect size (F(3, 5580) = 317.44, p =< .001, *η^2^_p_* = .15). Figure 1 shows bed and rise times according to age category for both questionnaires.

**Table 1:** Sex Differences in ISI Scores and Sleep Parameters (First Wave).


	TOTAL (*M, SD*)	MALE (*M, SD*)	FEMALE (*M, SD*)	*T(DF)*	*P*	*D*

**ISI pre**	6.00 (4.91)	5.51 (4.76)	6.24 (5.00)	–5.03 (5121)	<.001	–0.15

**ISI peri**	8.82 (5.91)	8.06 (5.89)	9.18 (5.89)	–6.68 (5538)	<.001	–0.19

**BT pre**	23:02 (1:10)	23:17 (1:16)	22:56 (1:06)	9.96 (5115)	<.001	0.30

**BT peri**	23:24 (1:29)	23:53 (1:36)	23:20 (1:25)	5.78 (5532)	<.001	0.17

**RT pre**	7:08 (1:18)	7:11 (1:19)	7:07 (1:18)	1.70 (5115)	.09	0.05

**RT peri**	7:53 (1:48)	7:52 (1:52)	7:54 (1:52)	–0.80 (5532)	.42	–0.02

**TST pre**	7:16 (1:05)	7:08 (1:04)	7:19 (1:04)	–6.01 (5115)	<.001	–0.18

**TST peri**	7:07 (1:22)	7:01 (1:20)	7:10 (1:23)	–3.75 (5532)	<.001	–0.11

**TIB pre**	8:05 (1:08)	7:54 (1:09)	8:11 (1:07)	–8.22 (5115)	<.001	–0.25

**TIB peri**	8:28 (1:25)	8:17 (1:29)	8:34 (1:23)	–7.05 (5532)	<.001	–0.20

**SOL pre**	0:19 (0:20)	0:18 (0:19)	0:20 (0:20)	–3.48 (5115)	<.001	–0.10

**SOL peri**	0:30 (0:33)	0:27 (0:32)	0:32 (0:34)	–4.83 (5532)	<.001	–0.14

**SE pre**	89.69 (9.94)	90.09 (9.85)	89.51 (9.99)	1.95 (5114)	.05	0.06

**SE peri**	84.30 (13.28)	85.10 (13.14)	83.94 (13.33)	3.07 (5523)	.002	0.09


*Note*: pre = pre-lockdown; peri = peri-lockdown; ISI = Insomnia Severity Index; BT = bedtime; RT = rise time; TST = total sleep time; TIB = time in bed; SOL = sleep onset latency; SE = sleep efficiency.

**Table 2:** Sex Differences in ISI Scores and Sleep Parameters (Second Wave).


	TOTAL (*M, SD*)	MALE (*M, SD*)	FEMALE (*M, SD*)	*T(DF)*	*P*	*D*

**ISI pre**	6.14 (5.05)	5.79 (5.12)	6.31 (5.02)	–2.19 (2098)	.03	–0.10

**ISI peri**	10.30 (6.54)	10.05 (6.80)	10.41 (6.41)	–1.26 (2398)	.21	–0.10

**BT pre**	22:58 (1:07)	23:09 (1:13)	22:53 (1:04)	5.05 (2098)	<.001	0.24

**BT peri**	23:06 (1:20)	23:14 (1:19)	23:02 (1:20)	3.56 (2397)	<.001	0.16

**RT pre**	7:04 (1:19)	6:59 (1:22)	7:06 (1:18)	–1.78 (2095)	.08	–0.08

**RT peri**	7:17 (1:32)	7:10 (1:30)	7:20 (1:33)	–2.51 (2395)	.01	–0.11

**TST pre**	7:14 (1:03)	7:04 (1:03)	7:19 (1:03)	–4.95 (2098)	<.001	–0.23

**TST peri**	6:52 (1:19)	6:43 (1:19)	6:57 (1:19)	–4.16 (2397)	<.001	–0.18

**TIB pre**	8:06 (1:05)	7:50 (1:08)	8:13 (1:02)	–7.40 (2095)	<.001	–0.35

**TIB peri**	8:10 (1:13)	7:55 (1:15)	8:18 (1:11)	–7.03 (2393)	<.001	–0.31

**SOL pre**	0:21 (0:19)	0:19 (0:18)	0:21 (0:20)	–2.18 (2098)	.03	–0.10

**SOL peri**	0:31 (0:31)	0:29 (0:29)	0:32 (0:31)	–2.54 (2398)	.01	–0.11

**SE pre**	89.35 (9.61)	90.06 (9.82)	89.05 (9.47)	2.24 (2094)	.03	0.11

**SE peri**	84.28 (12.76)	84.93 (13.05)	84.01 (12.58)	1.65 (2393)	.10	0.07


*Note*: pre = pre-lockdown; peri = peri-lockdown; ISI = Insomnia Severity Index; BT = bedtime; RT = rise time; TST = total sleep time; TIB = time in bed; SOL = sleep onset latency; SE = sleep efficiency.

**Table 3:** Age Differences in ISI Scores and Sleep Parameters (First Wave).


	TOTAL (*M, SD*)	AGE CATEGORIES				*F(DF)*	*P*	*η^2^_p_*

		<24Y (*M, SD*)	25–44Y (*M, SD*)	45–64Y (*M, SD*)	65Y+ (*M, SD*)			

**ISI pre**	6.00 (4.91)	6.91 (5.15)	5.76 (4.82)	6.08 (4.88)	5.31 (4.75)	12.95 (3, 5116)	<.001	.01

**ISI peri**	8.82 (5.91)	9.61 (6.13)	9.04 (5.89)	8.63 (5.85)	6.72 (5.32)	24.42 (3,5116)	<.001	.01

**BT pre**	23:02 (1:10)	23:28 (1:21)	22:55 (1:07)	22:57 (1:07)	23:20 (1:00)	55.93 (3,5160)	<.001	.03

**BT peri**	23:24 (1:29)	00:17 (1:51)	23:16 (1:28)	23:11 (1:12)	23:24 (1:08)	123.85 (3,5580)	<.001	.06

**RT pre**	7:08 (1:18)	7:58 (1:36)	7:00 (1:14)	6:50 (1:05)	7:45 (0:58)	189.50 (3,5160)	<.001	.10

**RT peri**	7:53 (1:48)	9:29 (2:03)	7:43 (1:42)	7:23 (1:30)	7:52 (1:07)	317.44 (3,5580)	<.001	.15

**TST pre**	7:16 (1:05)	7:38 (1:18)	7:17 (0:59)	7:02 (1:01)	7:19 (1:07)	58.11 (3,5160)	<.001	.03

**TST peri**	7:07 (1:22)	7:52 (1:27)	7:06 (1:19)	6:47 (1:18)	7:09 (1:15)	126.11 (3,5580)	<.001	.06

**TIB pre**	8:05 (1:08)	8:29 (1:29)	8:04 (1:00)	7:52 (1:04)	8:25 (1:10)	65.86 (3,5160)	<.001	.04

**TIB peri**	8:28 (1:25)	9:12 (1:28)	8:26 (1:25)	8:12 (1:20)	8:28 (1:13)	99.40 (3,5580)	<.001	.05

**SOL pre**	0:19 (0:20)	0:25 (0:25)	0:19 (0:19)	0:17 (0:17)	0:19 (0:19)	32.60 (3,5160)	<.001	.02

**SOL peri**	0:30 (0:33)	0:41 (0:43)	0:31 (0:33)	0:26 (0:29)	0:24 (0:26)	42.12 (3,5580)	<.001	.02

**SE pre**	89.69 (9.94)	89.97 (10.06)	90.28 (9.47)	89.41 (10.11)	86.76 (11.07)	14.09 (3,5158)	<.001	.01

**SE peri**	84.30 (13.28)	85.74 (13.30)	84.42 (13.11)	83.48 (13.55)	84.29 (12.84)	5.67 (3,5570)	.001	.003


*Note*: y = years; pre = pre-lockdown; peri = peri-lockdown; ISI = Insomnia Severity Index; TST = total sleep time; TIB = time in bed; SOL = sleep onset latency; SE = sleep efficiency.

**Table 4:** Age Differences in ISI Scores and Sleep Parameters (Second Wave).


	TOTAL (*M, SD*)	AGE CATEGORIES				*F(DF)*	*P*	*η^2^_p_*

		<24Y (*M, SD*)	25–44Y (*M, SD*)	45–64Y (*M, SD*)	65Y+ (*M, SD*)			

**ISI pre**	6.1 (5.1)	6.00 (4.70)	5.89 (4.80)	6.56 (5.47)	5.89 (5.11)	2.78 (3,2095)	.04	.004

**ISI peri**	10.3 (6.5)	9.96 (6.10)	10.33 (6.40)	10.66 (6.84)	8.75 (6.62)	3.74 (3,2095)	.01	.004

**BT pre**	22:58 (1:07)	23:12 (1:09)	22:54 (1:09)	22:53 (1:05)	23:16 (0:52)	9.11 (3,2099)	<.001	.01

**BT peri**	23:06 (1:20)	23:31 (1:28)	23:02 (1:24)	22:59 (1:10)	23:30 (1:07)	16.80 (3,2399)	<.001	.02

**RT pre**	7:04 (1:19)	7:57 (1:22)	6:59 (1:16)	6:42 (1:12)	7:32 (1:01)	78.42 (3,2096)	<.001	.10

**RT peri**	7:17 (1:32)	8:29 (1:40)	7:14 (1:30)	6:48 (1:16)	7:34 (1:13)	116.84 (3,2397)	<.001	.13

**TST pre**	7:14 (1:03)	7:53 (0:57)	7:16 (0:59)	6:57 (1:05)	7:02 (1:05)	61.59 (3,2099)	<.001	.08

**TST peri**	6:52 (1:19)	7:42 (1:13)	6:54 (1:16)	6:31 (1:17)	6:46 (1:14)	73.83 (3,2399)	<.001	.09

**TIB pre**	8:06 (1:05)	8:45 (1:06)	8:05 (1:02)	7:49 (1:01)	8:16 (1:06)	60.58 (3,2096)	<.001	.08

**TIB peri**	8:10 (1:13)	8:57 (1:14)	8:11 (1:11)	7:49 (1:06)	8:13 (1:10)	79.44 (3,2395)	<.001	.09

**SOL pre**	0:21 (0:19)	0:24 (0:20)	0:21 (0:19)	0:19 (0:19)	0:20 (0:19)	3.84 (3,2099)	.01	.01

**SOL peri**	0:31 (0:31)	0:36 (0:33)	0:31 (0:30)	0:30 (0:30)	0:28 (0:29)	3.90 (3,2400)	.01	.01

**SE pre**	89.35 (9.61)	90.04 (8.51)	89.98 (8.74)	88.80 (10.52)	85.51 (12.28)	8.43 (3,2095)	<.001	.01

**SE peri**	84.28 (12.76)	86.32 (11.14)	84.49 (12.67)	83.35 (13.22)	82.89 (12.76)	5.18 (3,2395)	.001	.01


*Note*: y = years; pre = pre-lockdown; peri = peri-lockdown; ISI = Insomnia Severity Index; TST = total sleep time; TIB = time in bed; SOL = sleep onset latency; SE = sleep efficiency.

### Effect of the pandemic on clinical insomnia

**Figure 2:**
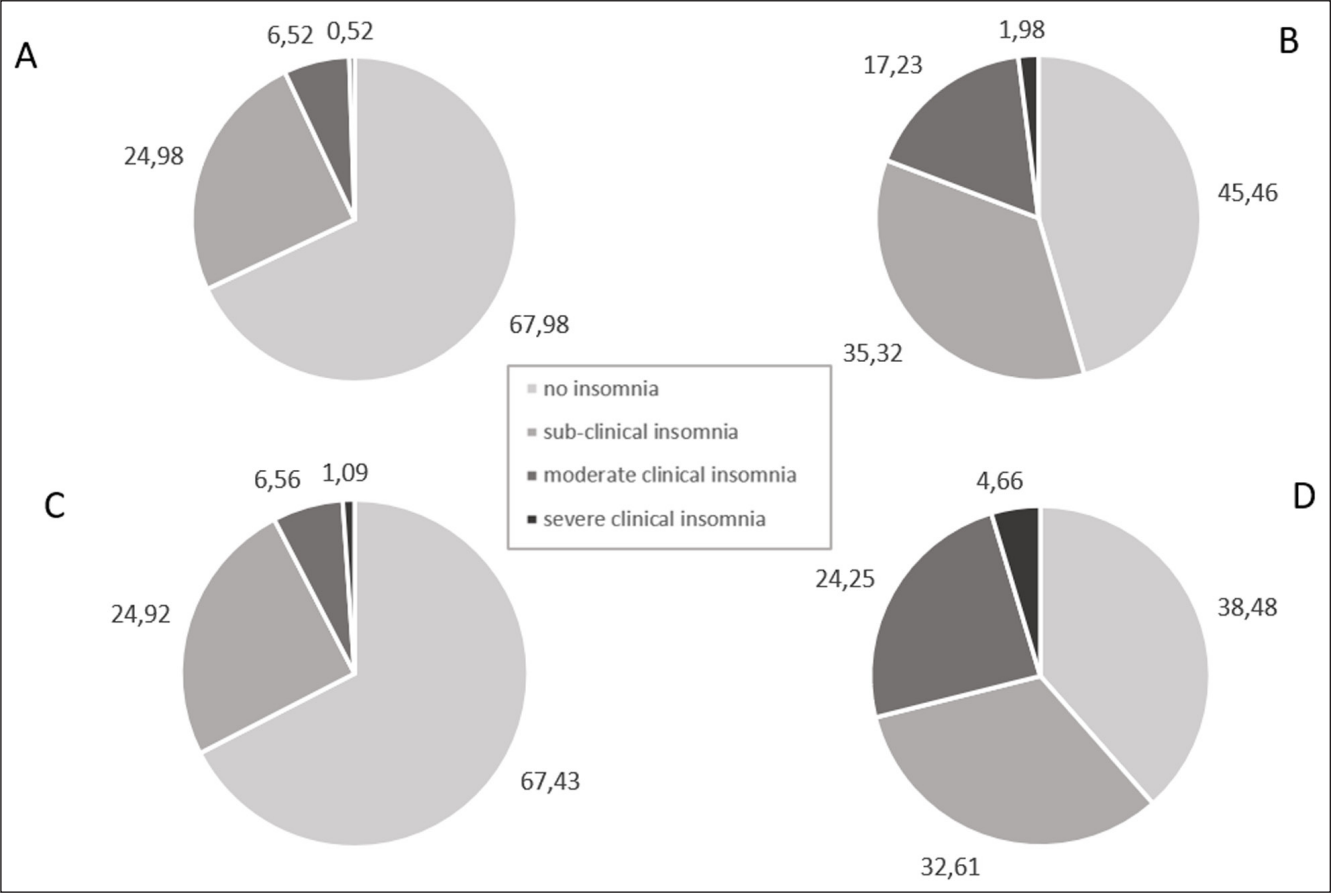
Prevalence of Clinical Insomnia. *Note*: (A) Pre-lockdown prevalences from the first questionnaire. (B) Peri-lockdown prevalences from the first questionnaire. (C) Pre-lockdown prevalences from the second questionnaire. (D) Peri-lockdown prevalences from the second questionnaire.

**Figure 3:**
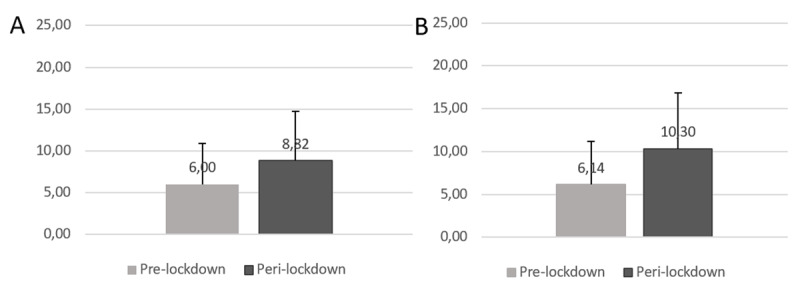
Insomnia Severity Index Scores. *Note*: (A) Results of the first questionnaire comparing the pre-covid situation with the first lockdown. (B) Results of the second questionnaire comparing the pre-covid situation with the second lockdown.

As can be seen in Figure 2, before the lockdown, 7.04% of the participants in our first study and 7.66% of the participants in our second study had clinical insomnia. This prevalence rose to 19.22% during the first lockdown and to 28.91% during the second. Figure 3 shows that the average ISI score was significantly higher during lockdown than before during both the first (*t*(5171) = –35.46, *p* =< .001, *d* = –0.49) and second wave (*t*(2102) = –33.31, *p* =< .001, *d* = –0.73). There was a significant difference in the distribution of ISI scores during both the first (*Q* = –29.14, *p* =< .001) and the second wave (*Q* = –24.88, *p* =< .001) in comparison with the pre-lockdown situation.

### Effect of the pandemic on self-reported sleep parameters

**Figure 4:**
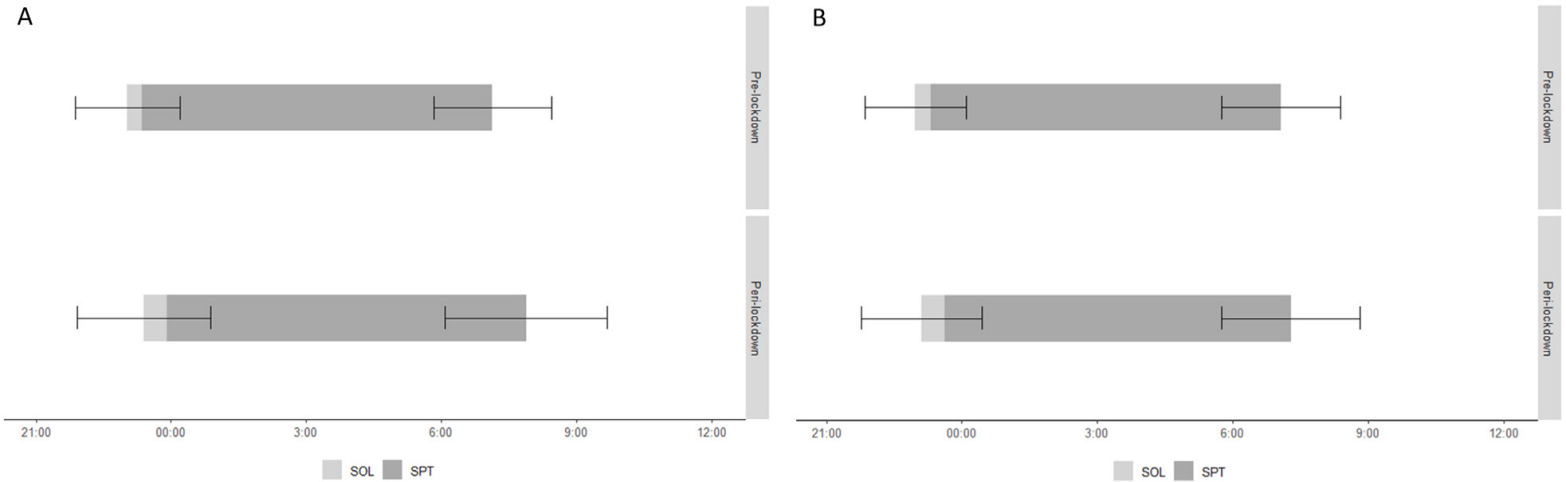
Effect of the Pandemic on Bedtime, Sleep Onset Latency and Rise Time. *Note*: SOL = sleep onset latency; SPT = sleep period time A) Results of the first questionnaire comparing the pre-covid situation with the first lockdown. (B) Results of the second questionnaire comparing the pre-covid situation with the second lockdown.

**Figure 5:**
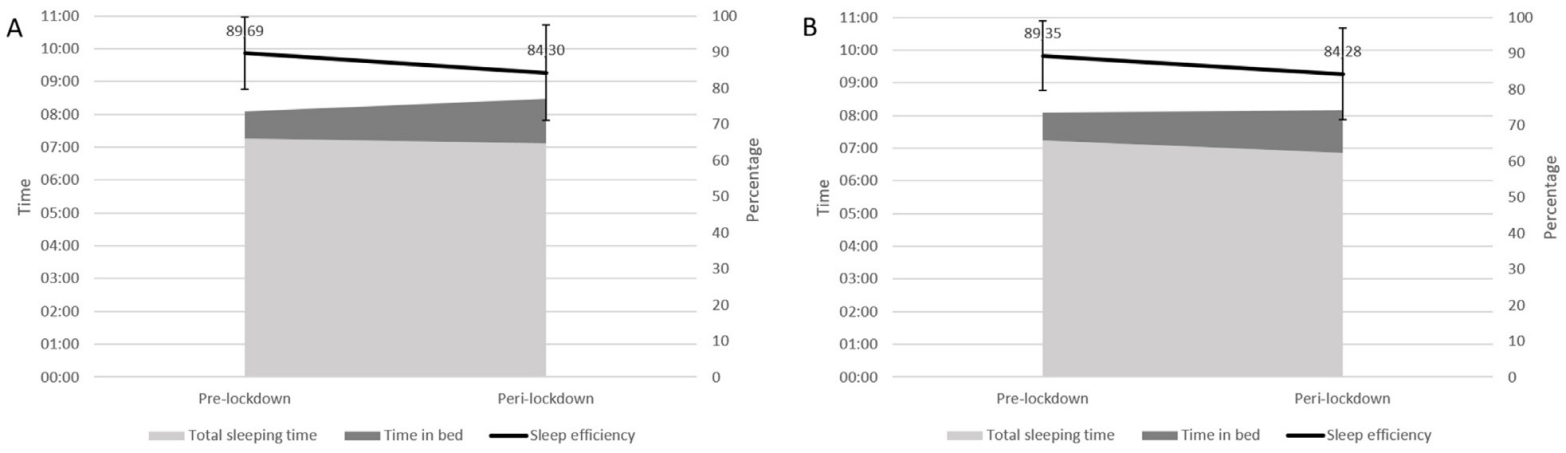
Effect of the Pandemic on Total Sleeping Time, Time in Bed and Sleep Efficiency. *Note*: (A) Results of the first questionnaire comparing the pre-covid situation with the first lockdown. (B) Results of the second questionnaire comparing the pre-covid situation with the second lockdown.

All results can be found in Table 5 for the first wave and in Table 6 for the second one. During both the first and the second lockdown, there was a significant delay in BT and RT in comparison with the pre-lockdown situation. During both lockdowns, there was also a significant increase in TIB and SOL in comparison with the pre-lockdown situation. Finally, we found a significant decrease in TST and SE during the two lockdowns in comparison with before the lockdown. Figure 4 and Figure 5 show these changes visually. Most effect sizes were small, except for the change in RT during the first lockdown (*t*(5165) = –39.30, *p* =< .001, *d* = –0.55) which had a medium effect size and the changes in TST during the first lockdown (*t*(5165) = 8.82, *p* =< .001, *d* = 0.12) and in BT (*t*(2101) = –7.97, *p* =< .001, *d* = –0.17) and TIB (*t*(2097) = –3.86, *p* =< .001, *d* = –0.08) during the second lockdown which had negligible effect sizes.

**Table 5:** Paired T-test Results for Sleep Parameters (First Wave).


	PRE-LOCKDOWN (*M, SD*)	PERI-LOCKDOWN (*M, SD*)	*T(DF)*	*P*	*D*

**Bed time**	23:02 (1:10)	23:24 (1:29)	–25.72 (5165)	<.001	–0.36

**Rise time**	7:08 (1:18)	7:53 (1:48)	–39.30 (5165)	<.001	–0.55

**Time in bed**	8:05 (1:08)	8:28 (1:25)	–21.61 (5165)	<.001	–0.30

**Total sleep time**	7:16 (1:05)	7:07 (1:22)	8.82 (5165)	<.001	0.12

**Sleep onset latency**	0:19 (0:20)	0:30 (0:33)	–30.72 (5165)	<.001	–0.43

**Sleep efficiency**	89.69 (9.94)	84.30 (13.28)	34.45 (5160)	<.001	0.48


**Table 6:** Paired T-test Results for Sleep Parameters (Second Wave).


	PRE-LOCKDOWN (*M, SD*)	PERI-LOCKDOWN (*M, SD*)	*T(DF)*	*P*	*D*

**Bed time**	22:58 (1:07)	23:06 (1:20)	–7.97 (2101)	<.001	–0.17

**Rise time**	7:04 (1:19)	7:17 (1:32)	–10.97 (2099)	<.001	–0.24

**Time in bed**	8:06 (1:05)	8:10 (1:13)	–3.86 (2097)	<.001	–0.08

**Total sleep time**	7:14 (1:03)	6:52 (1:19)	15.89 (2101)	<.001	0.35

**Sleep onset latency**	0:21 (0:19)	0:31 (0:31)	–20.23 (2102)	<.001	–0.44

**Sleep efficiency**	89.35 (9.61)	84.28 (12.76)	21.71 (2096)	<.001	0.47


## Discussion

The purpose of this study was to test if there were differences in the self-reported prevalence of clinical insomnia and in sleep habits in the Belgian population during the first and second lockdown in comparison with the pre-covid period.

### Effect of the pandemic on clinical insomnia

The number of individuals reporting symptoms of clinically significant insomnia as defined by an ISI score > 15, increased during the first lockdown and rose further during the second one. Our results of the first wave are in line with previous reports (Li et al., 2020; Lin et al., 2021; Marelli et al., 2021). Cox and Olatunji (2021) pointed out several aspects of the pandemic that form precipitating and perpetuating factors according to the 3P model of Insomnia such as fears of contracting the virus or financial strains, decreased contact with appropriately timed Zeitgebers and stimulus dyscontrol from increased time indoors (Spielman et al., 1987). This might explain why acute insomnia was triggered during the first wave of the pandemic in persons who already had predisposing factors and why the pandemic allowed for acute insomnia to become chronic during the second wave.

However, in contrast with Salfi et al. (2021), we found a larger effect of the pandemic on ISI scores during the second wave in comparison with the first. The authors did not give any explanation for the decrease in insomnia cases they observed. We hypothesize that this could be linked to the decrease in anxiety that they found between the two waves, which could again be linked to the difference in confinement strictness between Italy and Belgium. Hale et al. (2021) developed a stringency index, ranging from 0 to 100 and indicating how strict the measures taken by a country’s government were. During the first lockdown, Belgium soared to a stringency index of 81.48. During the second lockdown, the stringency index peaked at 75.93. Italy’s stringency index rose to 93.52 during the first confinement and decreased to 79.63 during the second study of Salfi et al (2021). Coming from such a strict confinement, the Italian population might have perceived their second confinement as less strict than the Belgian population did. This could have led to less anxiety during the second Italian confinement and thus to fewer insomnia cases, in comparison to the Belgian population. Another explanation could be methodological: as opposed to ours, the study of Salfi et al. (2021) included the same participants in both waves but did not include any form of baseline measurement (pre-pandemic situation). In the present study, because of pseudonymization, it remains unclear how much of our sample answered both questionnaires. Additionally, our frame of reference consists of an estimation of sleep habits and symptom severity before the occurrence of the pandemic.

### Effect of the pandemic on self-reported sleep parameters

During the first lockdown, surveyed individuals went on average 22 minutes later to bed and rose 45 minutes later. Their SE decreased by 5.39%: they spent 23 minutes longer in bed, but slept 9 minutes less, partially due to a SOL increased by 11 minutes. During the second lockdown, the average differences with the pre-lockdown situation were smaller, except for TST. People went 8 minutes later to bed and woke up 13 minutes later. Although their TIB only increased by 4 minutes, their TST decreased by 22 minutes. SOL increased by 10 minutes and SE decreased by 5.07%.

The results for the first lockdown corroborate previous findings: the delayed BT and RT are in line with most studies on this subject (Cellini et al., 2020, 2021; Gupta et al., 2020; Li et al., 2020; Majumdar et al., 2020; Marelli et al., 2021; Sinha et al., 2020; Wright et al., 2020), as does the increased TIB (Cellini et al., 2020, 2021; Li et al., 2020; Robillard et al., 2021). Remote working and online classes allowed workers and students to move their BT and RT at a time that supposedly better fitted their diurnal preferences, and to spend more time in bed. First, an increase in SOL was found in most of the studies that investigated this variable (Cellini et al., 2021; Gupta et al., 2020; Marelli et al., 2021; Robillard et al., 2021), which could be explained by an increase in psychological distress during the pandemic (Alimoradi et al., 2021; Huang et al., 2022; Zhang et al., 2022). However, current findings are mixed regarding reported sleep duration. Our results show a decreased TST (Gupta et al., 2020; López-Moreno et al., 2020; Robillard et al., 2021). This reduction in sleep duration could be explained by several factors such as increased affective symptomatology or a lack of physical activity during lockdown. In addition, altered perceptual factors inherent to insomnia symptomatology such as underestimation of sleep duration, might also explain an average decrease in reported TST. Furthermore, an increase in time spent in bed together with a decreased TST resulting in lower sleep efficiency, was also observed by Li and colleagues in a large-scale survey in COVID-free individuals (Li et al., 2020). Our findings also differ from those of the only other study that investigated sleep during the second lockdown. Unlike our results, Conte et al (2021) found no difference between pre-lockdown and second wave BT, RT, TIB and SOL. An easy explanation for this difference is the larger statistical power of our study, which included at least 15 times more participants than the study of Conte et al (2021).

The <24 years group had the greatest change in these parameters between pre- and peri-lockdown. They are also the only group who slept more during lockdown than before the lockdown. We hypothesize that younger individuals allowed to follow a sleep pattern that better suited their naturally more delayed circadian rhythm (Carskadon, 2011; Forquer et al., 2008) by going to bed and waking up later. They seem to have followed their weekend sleep schedule even on weekdays during lockdown. The sleep schedule differed for the 65+ years group the least between pre- and peri-lockdown. This could be explained by the fact that lockdown had a smaller impact on mostly retired individuals, who do not have to follow a certain sleep schedule related to their work.

The first Belgian confinement was more restrictive than the second one (full vs. partial) (Hale et al., 2021). As the effect sizes of BT, RT and TIB are larger for the full compared to the partial lockdown than for the partial, we may conclude that the latter had a smaller impact on people’s sleep schedules. This might be explained by the fact that outhouse work, school and leisure activities partially resumed, having individuals resorting to pre-lockdown sleep-wake schedules.

### Strengths and limitations

Both of our studies are characterized by a fairly large response rate considering the country’s population. Additionally, insomnia symptomatology was measured using a standardized, valid and reliable questionnaire (ISI) which is widely used in clinical practice. Since our participants answered the questionnaires twice, once for before the lockdown and once for during the lockdown, we did not have to rely on results from previous studies to compare both situations. However, this also means that there might be a recall bias and that participants’ view on their pre-lockdown sleep might be influenced by their view on their peri-lockdown sleep. Our comparison of the first and second lockdown is also limited by our study not being longitudinal. Our study also has some other limitations: measuring insomnia and sleep parameters was done through self-report questionnaires exclusively. Finally, although most of our results reached statistical significance, the magnitude of the observed effects was generally small.

### Implications

This significant increase in the prevalence of insomnia should be addressed through the availability of insomnia’s first-line treatment, cognitive behavioral therapy of insomnia (CBT-I) (Riemann et al., 2017). Furthermore, individuals aged less than 24 years reported delayed BT and RT compared to the other age groups, potentially being under greater risk of developing a delayed sleep-wake phase rhythm disorder during lockdown allegedly due to a to a lack of social and light entrainment and of daytime physical activity. These participants may thus require particular attention in terms of treatment (Sinha et al., 2020).

### Suggestions for further research

We suggest investigating the same parameters during the next waves to see the evolution of insomnia prevalence and sleep habits, with a particular focus on the adolescent and younger adult population. It would further be interesting to investigate potential causal relationships between the diurnal and the nocturnal symptoms of insomnia through network analysis.

## Conclusion

The number of persons with clinical insomnia rose during the first lockdown and further during the second. BT and RT were delayed during both lockdowns in comparison with the pre-lockdown situation. Furthermore, there was an increased TIB and SOL and decreased TST and SE during both confinements. Alterations in sleep habits were the most pronounced in the younger population (<24 yrs).

## Data availability statement

The data is available at the Open Science Framework: https://osf.io/tv2dw.

## Abbreviations

BT: Bedtime

CBT-I: Cognitive behavioral therapy for insomnia

ISI: Insomnia Severity Index

RT: Rise time

SE: Sleep efficiency

SOL: Sleep onset latency

TIB: Time in bed

TST: Total sleep time
